# Use of electronic cigarettes among Romanian university students: a cross-sectional study

**DOI:** 10.1186/s12889-015-1713-6

**Published:** 2015-04-11

**Authors:** Lucia Maria Lotrean

**Affiliations:** Iuliu Hatieganu University of Medicine and Pharmacy, Cluj-Napoca, Romania; Organization Pure Air, Bucharest, Romania

**Keywords:** Electronic cigarettes, University students, Romania

## Abstract

**Background:**

Because electronic cigarettes are relatively new, data on usage patterns and factors which influence them are sparse. Hence, this study aims at assessing awareness, beliefs about electronic cigarettes and experimentation with them among university students from Romania- a country where the sales and marketing of these products are widespread. Secondly, correlates of electronic cigarette experimentation will also be investigated.

**Methods:**

A cross-sectional study was performed by means of anonymous questionnaires among 480 students, aged 19–24, from Cluj-Napoca, Romania, between April-May 2013.

**Results:**

The results show that 92.5% of the students have heard about e-cigarettes; out of these, one quarter (53.3% of the smokers, 25% of the ex-smokers, 5.5% of the non-smokers) have tried electronic cigarettes at least once during lifetime. The results of the multinomial logistic regression point out that the correlates of electronic cigarette experimentation were: male gender, being a smoker of traditional cigarettes, having friends who experimented with electronic cigarettes, having stronger beliefs that electronic cigarettes could help them quit smoking and being less convinced that they are used only by smokers. The explained variance was 59%.

**Conclusions:**

The results underline the importance of addressing the issue of e-cigarette use through health education programs and regulatory interventions, since e-cigarettes are a reality faced by the Romanian youth.

## Background

Electronic cigarettes (e-cigarettes) are battery powered devices that convert nicotine containing liquid into a vapor that can be inhaled. In the last years they have been spread and advertised in several countries as a safe nicotine delivery device that can satisfy smokers’ addiction to both nicotine and smoking behaviors, reducing their risk of disease and increasing their chances of quitting smoking [1-3].

The opponents believe that e-cigarettes should undergo clinical trials to prove their safety and their efficacy in quitting smoking, like in the case of other therapeutic products. Concerns have been also raised that e-cigarettes will undermine efforts to denormalize smoking and, as they are novelty gadgets with a low perceived risk, they may be attractive to the youth, leading to nicotine addiction and subsequent tobacco use [1-4]. Because of these concerns, e-cigarettes were banned in some countries such as Canada and Australia [5]. However, in several European countries, such as Romania, e-cigarettes are legal. Moreover, they are heavily marketed and their sale and promotion is not restricted by any regulation [6,7].

Because e-cigarettes are relatively new, data on usage patterns are sparse [1-4]. Hence, this study aims at assessing awareness, beliefs about e-cigarettes and experimentation with them among university students from Romania. Secondly, correlates of e-cigarette experimentation will also be investigated.

## Method

### Design and instruments

A cross-sectional study was conducted between April-May 2013 in Cluj-Napoca, a town with approximately 330,000 inhabitants from North-West Romania. The study is part of a research grant which was reviewed and approved by the Ethics Committee of the University of Medicine and Pharmacy from Cluj-Napoca, Romania.

The study subjects were university students, aged 19–24, from 8 dorms belonging to the 4 main universities of the town (the total number of students attending these universities was 66150). A number of 60 girls and 60 boys living in the selected dorms were chosen from each university. The selection of students was made by randomly choosing two participants/room from 30 different rooms of each dorm (dorms had rooms with 2–4 students living in each room). The participants were informed that their participation in the study was voluntary and were asked to fill in an anonymous questionnaire. The refusal rate was 5.6%. Students who refused to participate were replaced with students from the same university, living in the same dorm. The study sample consisted of 480 students, representing 0.72% of the total number of students attending the 4 universities.

The questionnaire assessed socio-demographic characteristics (age, gender), awareness and beliefs about e-cigarettes, their use during one’s lifetime and reasons for using them, e-cigarettes use in the last month, intention to use them in the next year and experimentation with e-cigarettes by friends, parents and siblings. Smoking behaviour was evaluated, too; the persons who had been smoking traditional cigarettes in the last month were defined as smokers, those who had smoked in the past but not in the last month were considered ex-smokers and students who had never smoked traditional cigarettes were non-smokers. The intention to quit smoking in the future was also investigated among smokers.

### Analyses

The sample of this study is represented by students who have heard about e-cigarettes (N = 444).

Multinomial logistic regression analyses were performed to assess the correlates of e-cigarette experimentation among the whole study sample and among smokers. The dependent variable was experimentation with e-cigarettes (never vs tried e-cigarettes at least once during lifetime; reference were considered those who never experimented with e-cigarettes).The independent variables included as forced entry terms were: age, gender, smoking behavior (0 = non-smoker, 1 = ex-smoker, 2 = smoker), beliefs about e-cigarettes (E-cigarettes could help people quit smoking; E-cigarettes are less dangerous than traditional cigarettes; E-cigarettes are used only by smokers; the possibilities of answers were grouped in two categories - I totally disagree/I partially disagree/I do not know vs I totally agree/I partially agree), experimentation with e-cigarettes by friends, parents, siblings (no vs yes).

The analyses performed among smokers also included, as independent variables, the number of traditional cigarettes smoked per day (up to one pack/day vs more than one pack/day) and intention to quit traditional smoking in the next year (yes vs no).

Data analysis was performed with the SPSS-15 statistics program. Significant results are reported at p < 0.05.

## Results

### Awareness, beliefs and behavior

The results show that 33.8% of the students were smokers, 15.6% were ex-smokers, while 50.6% were non-smokers.

A percentage of 92.5% of the students (95.1% of the smokers, 96% of the ex-smokers and 89.7% of the non-smokers) reported having heard about e-cigarettes.

Table 1 shows that half of the study sample (students who were aware about e-cigarettes) believed that e-cigarettes were less dangerous than traditional cigarettes and two thirds of the students considered that e-cigarettes could help in quitting smoking. Half of the students believed that e-cigarettes were used only by smokers.

**Table 1:** **Awareness, beliefs and behavior related to e**-**cigarettes experimentation**

	**Total sample N = 444٭ %**	**Smokers N = 154٭ %**	**Ex**-**smokers N = 72٭ %**	**Non**-**smokers N = 218 %**
**Beliefs**				
**E-cigarettes are less dangerous**				
I totally agree/I partially agree	55.9	62.3	33.3	58.7
I do not know	35.8	35.8	50	31.2
I totally disagree/I partially disagree	8.3	1.9	16.7	10.1
**E-cigarettes can help smokers to quit**				
I totally agree/I partially agree	66.4	46.1	70.8	79.4
I do not know	22.5	33.8	20.8	15.1
I totally disagree/I partially disagree	11.1	20.1	8.3	5.5
**E-cigarettes are used only by smokers**				
I totally agree/I partially agree	48.9	51.3	50	46.8
I do not know	34.5	31.8	33.3	36.7
I totally disagree/I partially disagree	16.6	16.9	16.7	16.5
**Behavior**				
Used e-cigarettes at least once during lifetime	25.2	53.3	25	5.5
Used e-cigarettes in the last month	2.7	7.8	0	0
**Reasons for trying e-cigarettes among students who experimented with them**٭٭				
E-cigarettes are less dangerous	8	0	50	0
To reduce the number of traditional cigarettes	0	0	0	-
To quit smoking	23.2	31.7	0	-
Curiosity	62.5	65.9	50	58.3
Other friends also tried e-cigarettes	23.2	25.6	0	41.7
**Intention to use e-cigarettes in the next year**				
Definitely yes/probably yes	4.1	11.7	0	0
I do not know	8.6	19.5	4.2	2.3
Definitely no/probably no	87.3	68.8	95.8	97.7
**Social influences**				
Friends have tried e-cigarettes	59.7	67.5	66.7	51.8
Parents have tried e-cigarettes	6.3	7.8	4.2	6.0
Siblings have tried e-cigarettes	6.5	5.2	8.3	6.9

٭The study sample consisted of students who have ever heard about e-cigarettes.

٭٭The percentages are calculated for students who ever tried e-cigarettes (N = 82 among smokers, N = 18 among ex-smokers, N = 12 among non-smokers).

One quarter of the students who had heard about e-cigarettes (53.3% of the smokers, 25% of the ex-smokers and 5.5% of the non-smokers) declared that they had tried e-cigarettes at least once during their lifetime. 7.8% of the smokers declared having used e-cigarettes in the last month, but not the ex-smokers and non-smokers. The main reasons for ever trying e-cigarettes were curiosity and friends’ influences; one third of the smokers used them to quit smoking.

Almost 60% of the study sample declared having friends who had experimented with e-cigarettes, while 6.3% of the students had parents who had done this.

Intention to use e-cigarettes in the next year was declared by 11.7% of the smokers.

### Correlates of e-cigarette experimentation

Table 2 presents the factors associated with electronic cigarette experimentation. The results of the multinomial logistic regression analyses show that among the whole study sample male students, the smokers and those who had friends who had experimented with e-cigarettes were more likely to try e-cigarettes. Experimentation with e-cigarettes was also more frequent among students who were more convinced about the utility of e-cigarettes for smoking cessation and among those who had weaker beliefs that e-cigarettes are used only by smokers. These variables explained 59% of the e-cigarette related behavior.

**Table 2:** **Correlates of ever trying e-cigarettes: results of the multinomial logistic regression analyses** ^**a**^

	**Total sample (N=444)** ^**b**^		**Smokers (N=154)** ^**b**^	
**Independent variables**	**Exp(B)**	**95% CI**	**Exp(B)**	**95% CI**
Gender				
Female	1		1	
Male	6.29٭٭٭	2.98-13.24	11.99٭٭٭	3.35-42.90
E-cigarettes help quitting smoking				
I totally disagree/I partially disagree/I do not know	1		1	
I totally agree/I partially agree	2.95٭٭	1.46-5.95	12.09٭٭٭	3.30-44.22
E-cigarettes are less dangerous				
I totally disagree/I partially disagree/I do not know	1		1	
I totally agree/I partially agree	1.70٭٭٭	0.91-3.19	3.55٭	1.26-9.94
E-cigarettes are used only by smokers				
I totally disagree/I partially disagree/I do not know	1		1	
I totally agree/I partially agree	0.43٭٭	0.23-0.79	0.69	0.26-1.84
Smoking behavior				
Smoker	1			
Ex smoker	0.09٭٭٭	0.03-0.24		
Non-smoker	0.02٭٭٭	0.01-0.04		
Number of traditional cigarettes smoked/day				
Maximum 1 pack/day			1	
>1 pack/day			10.85٭٭٭	2.67-44.03
Intention to quit traditional smoking within the next year				
Yes			1	
No			0.374	0.12-1.15
Friends tried e-cigarettes				
No	1		1	
Yes	24.25٭٭٭	9.55-61.55	8.97٭٭٭	2.83-28.44
Parents tried e-cigarettes				
No	1		1	
Yes	0.51	0.15-1.70	0.92	0.21-3.87
Siblings tried e-cigarettes				
No	1		1	
Yes	0.41	0.07-2.24	2.30	0.07-6.92

^a^The reference were students who did not experimented with e-cigarettes.

^b^The study sample consisted of students who have ever heard about e-cigarettes.

٭p<0.05.

٭٭p<0.01.

٭٭٭p<0.001.

Among the smokers, the correlates of trying e-cigarettes were male gender, having friends who had tried e-cigarettes, smoking higher numbers of traditional cigarettes per day and having stronger beliefs that e-cigarettes are less dangerous than traditional cigarettes and that they could help in quitting smoking. Intention to quit traditional smoking in the next year was not statistically significant associated with the experimentation of e-cigarettes. The explained variance was 55%.

## Discussion

This is the first Romanian study and one of the few European studies investigating the use of e-cigarettes among young people [1-3].

The results show a high awareness and a substantial experimentation with e-cigarettes among university students in Romania - a country where the sales and marketing of these products are widespread, with no restrictions and regulations. Almost all of the students have heard about e-cigarettes, while half of the smokers, one quarter of the ex-smokers, but also 5.5% of the non-smokers have tried e-cigarettes during their lifetime. These data are similar with those from another study performed among university students from Poland [7]. On the other hand, the use of e-cigarettes in the month previous the survey was low, only 7.8% of the smokers used them, while the ex-smokers and non-smokers did not.

Furthermore, the study focused on identifying factors associated with e-cigarette experimentation. Similar with the Polish study [7], among our study sample, electronic cigarette experimentation was more frequent among smokers than non-smokers, while boys were more tempted to try these products than girls.

The main reasons for trying e-cigarettes declared by the students, independent of their smoking status, was curiosity to try this new product; future studies should investigate if this type of experimentation is an isolated event or if it could lead to a frequent use.

The influence coming from friends was also an important reason for trying e-cigarettes mentioned by the students; the logistic regression analyses confirmed that experimentation with e-cigarettes was related to friends’ experimentation with e-cigarettes.

One third of the smokers who used e-cigarettes declared that they had done this to quit smoking. The logistic regression analyses also underlined that this use of e-cigarettes among smokers was more frequent among those who smoked more traditional cigarettes/day and had stronger beliefs that e-cigarettes could help in quitting smoking. Nevertheless, similar with other studies [1,8], intention to quit smoking soon was not associated with experimentation with e-cigarettes.

The study has several limitations. First, it involved only students from one big town of Romania. The study did not include a national representative sample, which limits the generalization of the results beyond its sample. Second, the sample size didn’t allow for the analysis of the data separately for boys and girls. Third, due to the cross-sectional design, the identification of causal relationship is not possible. Future studies should include a national representative sample and should focus on gender differences, too.

## Conclusions

This study offers information about usage patterns of e-cigarettes and the factors which influence experimentation with e-cigarettes among Romanian university students.

The results underline the importance of addressing the issue of e-cigarette use through health education programs and regulatory interventions, since e-cigarettes are a reality faced by the Romanian youth.
